# fastfrechet: An R package for fast implementation of Fréchet regression with distributional responses

**DOI:** 10.21105/joss.07925

**Published:** 2025-05-01

**Authors:** Alexander Coulter, Rebecca Lee, Irina Gaynanova

**Affiliations:** 1 Department of Statistics, Texas A&M University, United States; 2 Department of Biostatistics, University of Michigan, United States

## Abstract

Distribution-as-response regression problems are gaining wider attention, especially within biomedical settings where observation-rich patient specific data sets are available, such as feature densities in CT scans (Petersen et al., 2021), actigraphy (Ghosal et al., 2023), and continuous glucose monitoring (Coulter et al., 2024; Matabuena et al., 2021). To accommodate the complex structure of such problems, Petersen & Müller (2019) proposed a regression framework called *Fréchet regression* which allows non-Euclidean responses, including distributional responses. This regression framework was further extended for variable selection by Tucker et al. (2023), and Coulter et al. (2024) developed a fast variable selection algorithm for the specific setting of univariate distributional responses equipped with the 2-Wasserstein metric (*2-Wasserstein space*). We present fastfrechet, an R package providing fast implementation of these Fréchet regression and variable selection methods in 2-Wasserstein space, with resampling tools for automatic variable selection. fastfrechet makes distribution-based Fréchet regression with resampling-supplemented variable selection readily available and highly scalable to large data sets, such as the UK Biobank (Doherty et al., 2017).

## Statement of Need

Fréchet regression with variable selection is currently not implemented by any software package, available only through the Supplementary Material of Tucker et al. (2023) (hereafter “Tucker materials”). The Tucker algorithm can be slow in many applications, for example taking 1.5 hours to run on a modest 207 patient, 34 covariate data set size from the HYPNOS CGM cohort; applying resampling methods like complementary pairs stability selection would be infeasible, taking upward of several CPU-days (Coulter et al., 2024). Implementation of the Fréchet regression problem in 2-Wasserstein space (i.e. without variable selection) is supported by the Tucker materials, and by two R packages: WRI (Liu et al., 2022) and frechet (Chen et al., 2023). These packages face certain practical limitations. For instance, WRI requires continuous distributions, and does not allow user-specified constraints for the distribution support. frechet offers more flexibility in user specifications, but its solver for Fréchet regression may not accurately satisfy constraints and is comparatively slow (i.e. takes upward of 10,000× longer than fastfrechet), as we show in the next section.

The fastfrechet package addresses these limitations by providing a fast, scalable, and user-friendly implementation of both Fréchet regression and variable selection for 2-Wasserstein space, based on the work of Coulter et al. (2024). The Fréchet regression solver features a customized dual active-set algorithm, inspired by Arnström et al. (2022), which ensures both computational efficiency and accuracy while accommodating user-specified support constraints. To support variable selection, it is also the first Fréchet regression solver to incorporate an auxiliary weighting scheme. In this scheme, the covariate-dependent weights that determine each observation’s influence can be modified using a user-supplied vector λ (Tucker et al., 2023), which specifies which covariates are excluded from the weight construction. The package incorporates resampling tools to enhance automatic variable selection, including cross-validation described in Tucker et al. (2023) and stability selection described in Coulter et al. (2024).

## Performance Comparisons to Existing Implementations

We illustrate the performance of fastfrechet against existing implementations with simulated covariate-dependent distributional responses. The included function generate_zinbinom_qf simulates *n* zero-inflated negative binomial (**zinbinom**) distributions (we choose *n* = 100), represented as quantile functions evaluated on a shared *m*-grid in (0, 1) (we choose *m* = 100), and dependent on the first 4 of *p* ≥ 4 covariates (we choose *p* = 10). We utilize the R package microbenchmark (Mersmann, 2024) to calculate run times, and report median times for each method (Fréchet regression, variable selection) from 15 iterations; all computations were performed on an Apple M1 Max chip. For a more detailed description and to replicate the specific simulation settings used in this manuscript, see the accompanying performanceExample-fastfrechet vignette.

### The Fréchet Regression Problem

fastfrechet provides a solver for the Fréchet regression problem for 2-Wasserstein space, with optional lower and upper support constraints on the underlying distributions. Since **zinbinom** distributions are non-negative, we fix lower = 0 and upper = Inf (or some suitably large number, as applicable). The regression outputs are fitted quantile functions, which should be monotone non-decreasing and obey support constraints. The fastfrechet implementation is a customization of the dual active-set method of Arnström et al. (2022). (See the accompanying monotoneQP-fastfrechet vignette for full algorithm description.)

**Figure 1:**
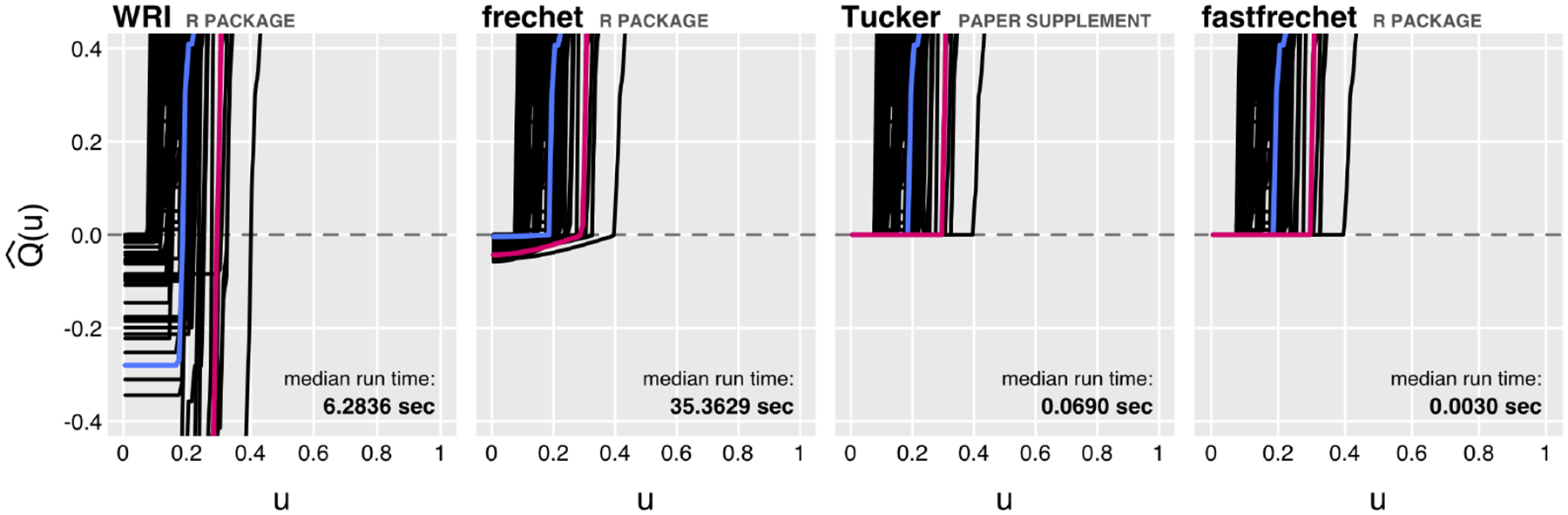
Fitted Fréchet regression quantile functions (zoomed in around zero) and median run times for fastfrechet and other implementations. Fitted quantile functions below zero violate known lower support constraints.

Figure 1 illustrates the speed and accuracy of Fréchet regression implemented in fastfrechet against the WRI, frechet, and Tucker materials implementations. WRI does not accept known support bounds as input, and fitted responses correspondingly violate the zero lower bound; frechet solutions only approximately satisfy the lower bound. The Tucker materials implementation finds numerically accurate solutions, but fastfrechet accomplishes this in a fraction the time. Applying support constraints *post hoc*, the solutions from WRI and frechet solutions remain sub-optimal minimizers of the Fréchet regression objective function. (See the accompanying performanceExample-fastfrechet vignette.)

### The Variable Selection Problem

The R package fastfrechet implements variable selection for Fréchet regression, specifically in 2-Wasserstein space. Variable selection comprises finding optimal weight vector $\hat{\lambda }\;\in \;{\mathbb{R}}^{p}$ that satisfies a *τ*-simplex constraint, given hyperparameter *τ* > 0. In 2-Wasserstein space, $\hat{\lambda }$ essentially minimizes an *L*^2^ norm between weighted Fréchet regression outputs $\hat{Q}\left(\hat{\lambda }\right)$ and the raw data ***Y***. (See the accompanying intro-fastfrechet vignette for a detailed exposition.) fastfrechet implements the second-order geodesic descent algorithm developed by Coulter et al. (2024), with two modifications. First, the implementation uses the custom dual active-set method mentioned in the previous subsection. The active set defining the weighted Fréchet regression solution $\hat{Q}\left(\lambda^{t}\right)$ for iterate $\lambda^{t}$ serves as a warm start for iterate **λ**^*t*+1^, reducing computation time. Second, the implementation allows the user to specify an impulse parameter, which implements momentum-based geodesic descent.

**Figure 2:**
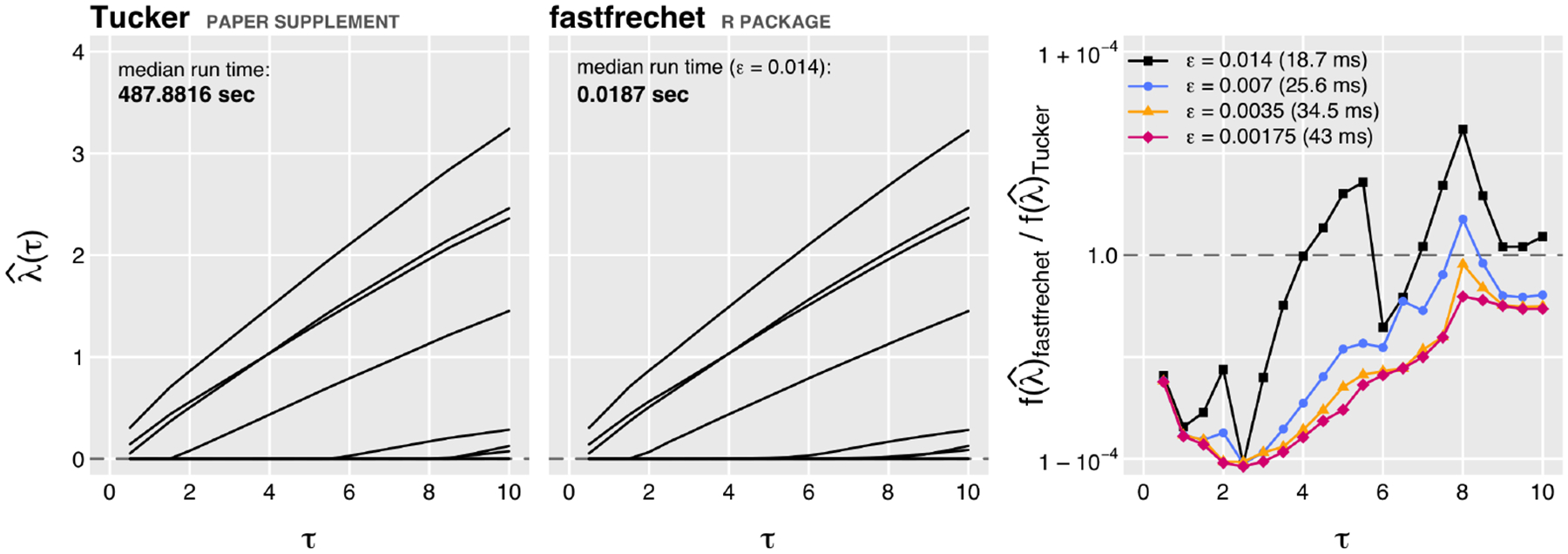
(**left, center**) Variable selection solution paths $\hat{\lambda }\left(\tau \right)$ across *τ* = {0.5,1,…,10} and median run times for Tucker materials and fastfrechet. (**right**) Relative optimization accuracy of fastfrechet and Tucker materials variable selection, and median fastfrechet run times, using different error tolerance values. Points below 1.0 indicate fastfrechet solutions minimize the objective function better.

Figure 2 illustrates the speed and accuracy of variable selection implemented in fastfrechet against the Tucker materials implementation, across sequence of hyperparameter values *τ* ∈ {0.5,1.0,⋯,10.0}. We hand-select fastfrechet error tolerance parameter *ε* = 0.014, which gives solutions $\hat{\lambda }\left(\tau \right)$ minimizing the objective function approximately as well as solutions from the other method “as-is”. fastfrechet is upward of 20,000× faster to obtain these comparable solutions. Decreasing the fastfrechet error tolerance parameter increases optimization accuracy with modest increases in computation time.

## References

[R1] Arnström D, Bemporad A, & Axehill D (2022). A Dual Active-Set Solver for Embedded Quadratic Programming Using Recursive LDL’ Updates. IEEE Transactions on Automatic Control, 67(8), 4362–4369. 10.1109/TAC.2022.3176430

[R2] Chen Y, Zhou Y, Chen H, Gajardo A, Fan J, Zhong Q, Dubey P, Han K, Bhattacharjee S, Alexander P, & Müller H-G (2023). Frechet: Statistical Analysis for Random Objects and Non-Euclidean Data. 10.32614/cran.package.frechet

[R3] Coulter A, Aurora RN, Punjabi NM, & Gaynanova I (2024). Fast variable selection for distributional regression with application to continuous glucose monitoring data. arXiv Preprint arXiv:2403.00922. 10.48550/ARXIV.2403.00922 PMC12700301 41393224

[R4] Doherty A, Jackson D, Hammerla N, Plötz T, Olivier P, Granat MH, White T, Van Hees VT, Trenell MI, Owen CG, Preece SJ, Gillions R, Sheard S, Peakman T, Brage S, & Wareham NJ (2017). Large Scale Population Assessment of Physical Activity Using Wrist Worn Accelerometers: The UK Biobank Study. PLOS ONE, 12(2), e0169649. 10.1371/journal.pone.0169649 28146576 PMC5287488

[R5] Ghosal R, Varma VR, Volfson D, Hillel I, Urbanek J, Hausdorff JM, Watts A, & Zipunnikov V (2023). Distributional data analysis via quantile functions and its application to modeling digital biomarkers of gait in Alzheimer’s Disease. Biostatistics, 24(3), 539–561. 10.1093/biostatistics/kxab041 36519565 PMC10544806

[R6] Liu X, Zhang C, Coleman M, & Petersen A (2022). WRI: Wasserstein Regression and Inference. 10.32614/cran.package.wri

[R7] Matabuena M, Petersen A, Vidal JC, & Gude F (2021). Glucodensities: A new representation of glucose profiles using distributional data analysis. Statistical Methods in Medical Research, 30(6), 1445–1464. 10.1177/0962280221998064 33760665 PMC8189016

[R8] Mersmann O (2024). Microbenchmark: Accurate Timing Functions. 10.32614/cran.package.microbenchmark

[R9] Petersen A, Liu X, & Divani AA (2021). Wasserstein F-tests and confidence bands for the Fréchet regression of density response curves. The Annals of Statistics, 49(1), 590–611. 10.1214/20-AOS1971

[R10] Petersen A, & Müller H-G (2019). Fréchet regression for random objects with Euclidean predictors. The Annals of Statistics, 47(2), 691–719. 10.1214/17-AOS1624

[R11] Tucker DC, Wu Y, & Müller H-G (2023). Variable Selection for Global Fréchet Regression. Journal of the American Statistical Association, 118(542), 1023–1037. 10.1080/01621459.2021.1969240

